# Activation of signal transducer and activator of transcription 3 (STAT3) signaling in EGFR mutant non-small-cell lung cancer (NSCLC)

**DOI:** 10.18632/oncotarget.17625

**Published:** 2017-05-04

**Authors:** Carles Codony-Servat, Jordi Codony-Servat, Niki Karachaliou, Miguel Angel Molina, Imane Chaib, Jose Luis Ramirez, Maria de los Llanos Gil, Flavio Solca, Trever G. Bivona, Rafael Rosell

**Affiliations:** ^1^ Pangaea Oncology, Barcelona, Spain; ^2^ Instituto Oncológico Dr Rosell (IOR), University Hospital Sagrat Cor, Barcelona, Spain; ^3^ Institut d’Investigació en Ciències Germans Trias i Pujol, Badalona, Spain; ^4^ Institut Català d’Oncologia, Hospital Germans Trias i Pujol, Badalona, Spain; ^5^ Instituto Oncológico Dr Rosell (IOR), Quirón-Dexeus University Institute, Barcelona, Spain; ^6^ Boehringer Ingelheim RCV GmbH and Co. KG, Vienna, Austria; ^7^ UCSF Helen Diller Family Comprehensive Cancer Center, San Francisco, United States

**Keywords:** lung cancer, EGFR, STAT3, afatinib, resistance

## Abstract

Gefitinib, erlotinib or afatinib are the current treatment for non-small-cell lung cancer (NSCLC) harboring an activating mutation of the epidermal growth factor receptor (EGFR), but less than 5% of patients achieve a complete response and the median progression-free survival is no longer than 12 months. Early adaptive resistance can occur as soon as two hours after starting treatment by activating signal transducer and activation of transcription 3 (STAT3) signaling. We investigated the activation of STAT3 in a panel of gefitinib-sensitive EGFR mutant cell lines, and gefitinib-resistant PC9 cell lines developed in our laboratory. Afatinib has great activity in gefitinib-sensitive as well as in gefitinib-resistant EGFR mutant NSCLC cell lines. However, afatinib therapy causes phosphorylation of STAT3 tyrosine 705 (pSTAT3_Tyr705_) and elevation of STAT3 and RANTES mRNA levels. The combination of afatinib with TPCA-1 (a STAT3 inhibitor) ablated pSTAT3_Tyr705_ and down-regulated STAT3 and RANTES mRNA levels with significant growth inhibitory effect in both gefitinib-sensitive and gefitinib-resistant EGFR mutant NSCLC cell lines. Aldehyde dehydrogenase positive (ALDH^+^) cells were still observed with the combination at the time that Hairy and Enhancer of Split 1 (HES1) mRNA expression was elevated following therapy. Although the combination of afatinib with STAT3 inhibition cannot eliminate the potential problem of a remnant cancer stem cell population, it represents a substantial advantage and opportunity to further prolong progression free survival and probably could increase the response rate in comparison to the current standard of single therapy.

## INTRODUCTION

The epidermal growth factor receptor (EGFR)-directed tyrosine kinase inhibitors (TKIs) gefitinib, erlotinib and afatinib are approved therapies for non-small-cell lung cancer (NSCLC) harboring activating mutations in the EGFR kinase [1–3]. Gefitinib and erlotinib are reversible inhibitors that target the ATP-site of the kinase. In contrast, afatinib, a second generation irreversible epidermal growth factor family of receptor tyrosine kinases (ErbB) blocker, not only targets the ATP-site of the receptor, but also covalently binds to cysteine 797 (C797) of EGFR which allows prolonged inhibition of EGFR phosphorylation, even in the presence of a T790M secondary mutation [4]. Afatinib also forms a covalent bond with the cysteine residue of HER2 (C805) and ErbB-4 (C803) and thus expands the inhibitory scope to the entire ErbB receptor family [5]. Resistance to EGFR TKIs arises rapidly and is attributed to the acquisition of a secondary T790M mutation between the ATP site of the receptor. Other common mechanisms of resistance have been reported, such as MET amplification and AXL overexpression [6]. In addition, selective irreversible inhibitors are highly active against the T790M mutant, but their efficacy can be limited by the acquired C797 mutation [7]. Intriguingly, in mouse models of EGFR (L858R/T790M/C797S) driven lung cancer, a new compound that targets selected drug-resistant EGFR but spares the wild-type receptor is effective in combination with cetuximab, an antibody that blocks EGFR dimerization and thus keeps the receptor in its inactive form [8]. Of interest is the fact that the combination of afatinib with 5-fluorouracil or pemetrexed inhibited the proliferation of NSCLC cells *in vitro*, whereas the combination of gefitinib with either of the two drugs was antagonistic. Afatinib induced downregulation of thymidylate synthase in the gefitinib-resistant NSCLC cells [9].

Afatinib was effective at lower concentrations than gefitinib in PC9 cells (derived from an untreated Japanese patient with lung adenocarcinoma carrying an exon 19 deletion [10]). Compared to gefitinib, afatinib also significantly prolonged survival of transgenic mice harboring an exon 19 deletion [11]. These findings mirror the results of the LUX-Lung 7 trial, where progression-free survival (PFS) was longer with afatinib in comparison to gefitinib, with a hazard ratio of 0.73, *p* = 0.017 [12]. Analysis of PFS according to mutation type shows a PFS of 12.7 months for afatinib and 11 months for gefitinib (hazard ratio 0.76) [12]. The PFS curves separate more significantly with time, commencing at the median PFS [12]. In addition, the proportion of patients achieving an objective response with afatinib was higher than with gefitinib (70% and 56% respectively; ratio 1.87, *p* = 0.008) [12], but only 1% of patients treated with either afatinib or gefitinib obtained a complete response [12].

In PC9 or gefitinib-resistant PC9 cells, signal transducer and activator of transcription (STAT3) phosphorylation is not inhibited with gefitinib or afatinib, in comparison to the down-regulation of AKT and ERK phosphorylation [11]. EGFR mutant cells show early activation of BCL-2/BCL-XL survival signaling via activation of STAT3 [13]. By day nine of erlotinib inhibition in the HCC827 and PC9 cells, there were cell subpopulations (early sur*vivo*rs that evaded and survived erlotinib therapy). Tumor cells that survived up to 6–9 days of erlotinib treatment signaled independently of EGFR and MET with early STAT3 reactivation [13]. Blockage of STAT3 phosphorylation on the critical tyrosine residue 705 (pSTAT3_Tyr705_) by niclosamide or TPCA-1 or depletion of STAT3 by RNA interference in HCC827 cells, reverts erlotinib resistance [14, 15]. Although STAT3 is activated by EGFR mutations [16, 17], other receptor and non-receptor tyrosine kinases such as the interleukin-6 (IL-6)/gp130 receptor can also stimulate STAT3 activation [18]. The levels of IL-6 mRNA were elevated in several EGFR mutant cell lines, including 11–18 and H1975. The mRNA levels of other IL-6 cytokines, such as oncostatin M, leukemia inhibitory factor, IL-11 and ciliary neurotropic factor were not detected [18]. Such findings show that IL-6 is secreted from these EGFR mutant cells and leads to activation of the gp130/JAK/STAT3 signaling pathway [18]. Introduction of activated EGFR into immortalized breast cells leads to carcinogenesis, IL-6 expression and STAT3 phosphorylation, which were all inhibited with a pan-JAK inhibitor or gp130 blockade [18]. Treatment of EGFR mutant cell lines with a pan-JAK inhibitor abrogated STAT3 and inhibited tumorigenesis while neither EGFR inhibitor nor dasatinib (Src kinase inhibitor) had any effect on STAT3 activity [18]. Afatinib was also shown to activate IL-6R/JAK1/STAT3 signaling pathway via interaction with fibroblasts and autocrine IL-6 secretion in H1975 and PC9 gefitinib-resistant cells [19]. Blockade of IL-6R/JAK1 significantly increased the sensitivity to afatinib [19]. Pre-incubation of PC9 cells with erlotinib-treated conditioned media increased transcriptional activities of stress/immune response-associated factors, such as STAT3, interefron regulatory factor-1 and interferon gamma. Erlotinib directly increased pSTAT3_Tyr705_ in EGFR mutant cells, but did not affect STAT3 in EGFR wild type cells [20]. Lung cancer stem cells have elevated aldehyde dehydrogenase (ALDH) activity and erlotinib increased the ALDH stem-like cells in EGFR mutant cancer cells [21, 22].

We have shown that gefitinib or the third generation EGFR TKI osimertinib cannot inhibit STAT3 and YAP in EGFR mutant cell lines [23]. In the current study, we examined EGFR mutant cell lines, including gefitinib-resistant PC9 cell lines, for the effect of afatinib in combination with TPCA-1, in inhibiting tumor growth proliferation and influencing signaling pathways that could down regulate STAT3. TPCA-1 (2-[(aminocarbonyl)amino]-5-(4-fluorophenyl)-3-thiophenecarboxamide), is a non-peptidic small molecule inhibitor of the inhibitor of κB kinase-2 (IKK2) [24], and the Src homology 2-domain of STAT3 [15]. The afatinib with TPCA-1 combination effectively down-regulated pSTAT3_Tyr705_, with a substantial growth inhibitory effect in gefitinib sensitive cell lines, as well as those in gefitinib resistant cell lines, with or without the T790M mutation.

## RESULTS

### Comparison of the sensitivity/resistance profile of EGFR mutant NSCLC cell lines to afatinib

**Figure 1:**
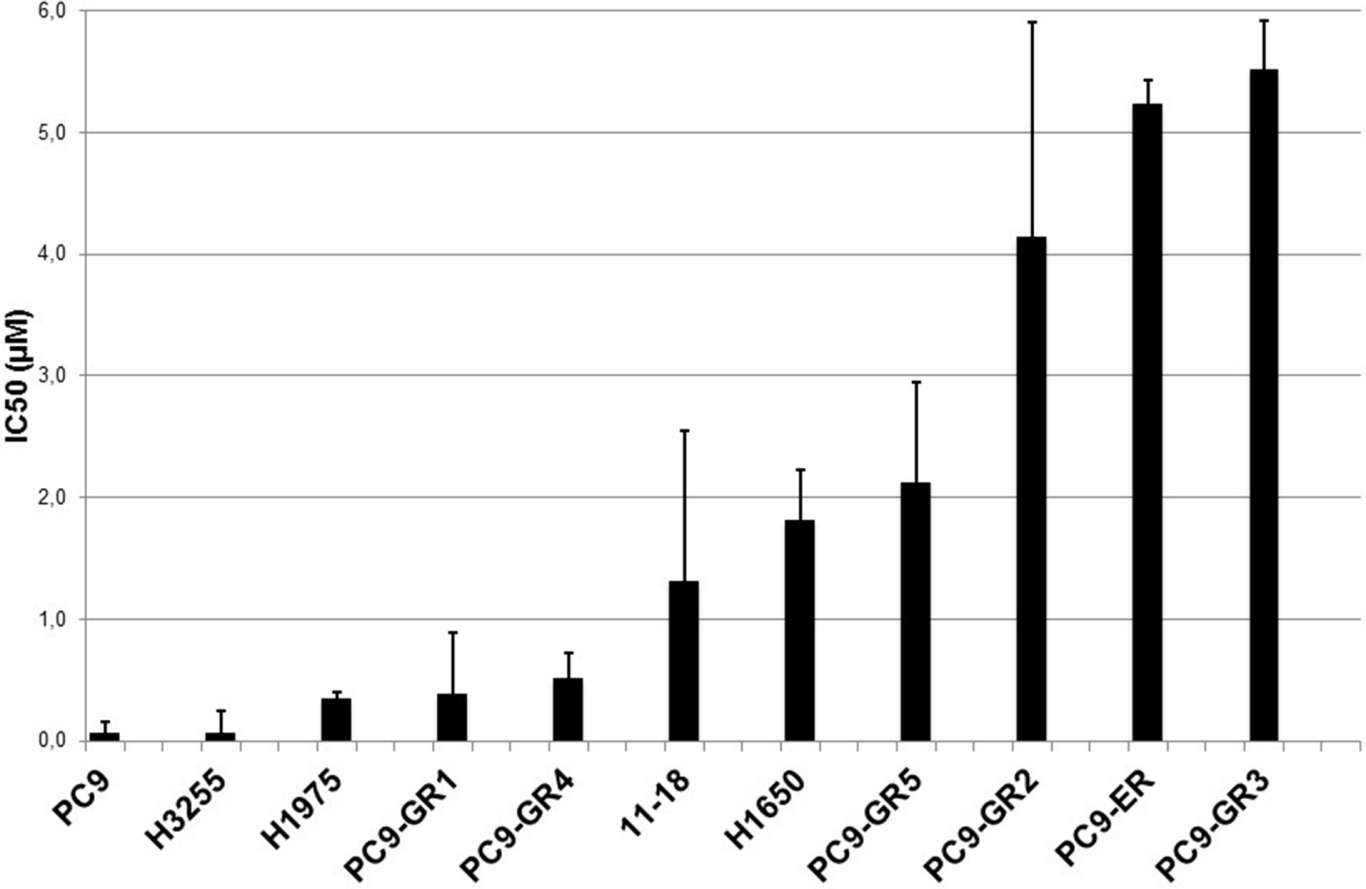
*In vitro* sensitivity to afatinib Eleven cell lines with IC50 values represented in μM. Error bars indicate the standard error based on multiple experiments.

We first assessed the activity of afatinib in 11 human EGFR mutant NSCLC cell lines by using the MTT (tetrazolium-based semiautomated colorimetric 3(4,5-dimethylthiazol-2-yl)-2,5-diphenyltetrazolium bromide) cell proliferation assay (Figure 1 and Table 1). The activity of the covalent inhibitor afatinib was compared with the activity of the two reversible EGFR TKIs gefitinib and erlotinib. The gefitinib and erlotinib sensitive PC9 cell line that harbors the EGFR exon 19 deletion was exquisitely sensitive to afatinib exposure (Figure 1 and Table 1). H3255 and 11–18 cells that carry the EGFR exon 21 L858R mutation were more sensitive to gefitinib compared to afatinib. EGFR is amplified approximately 11-fold in H3255 [25]. Among the three EGFR TKIs, afatinib had by far the highest molar potency in the H1975 cell line that carries the L858R/T790M double mutant EGFR.

**Table 1:** Characterization of EGFR mutant NSCLC cell lines and sensitivity to afatinib, erlotinib and gefitinib

Cell lines	EGFR status	IC50 (μM)		
		Afatinib	Erlotinib	Gefitinib
PC9	Del19	0.003	0.045	0.040
H3255	L858R *(EGFR amplification)*	0.0057	--	0.002
PC9-GR1	Del19, T790M (25%)	0.323	4.828	11.140
PC9-GR2	Del19	4.082	25.055	21.735
PC9-GR3	Del19	5.457	33.743	15.752
PC9-GR4	Del19, T790M (38%)	0.452	3.842	6.271
PC9-GR5	Del19	2.062	21.840	18.125
PC9-ER	Del19	5.176	28.598	14.356
11-18	L858R	1.256	--	0.491
H1650	Del19 *(PTEN loss/low BIM expression)*	1.759	--	13.665
H1975	L858R/T790M	0.287	2.514	9.221

We have generated six EGFR TKI-resistant cell lines by treating EGFR TKI-sensitive PC9 cells with increasing concentrations of gefitinib (GR1-5) or erlotinib (ER). The half-maximal inhibitory concentration (IC50) for afatinib, gefitinib and erlotinib of parental PC9 cells was in the nanomolar range compared to 4–34 μM in the resistant cell lines. Sequencing analyses revealed that all six cell lines retained the EGFR exon 19 deletion (Table 1), while the T790M mutation emerged in two of them (PC9-GR1 and PC9-GR4 at allelic fractions of 25 and 38% respectively). Gene expression analysis by TaqMan based quantitative Reverse Transcription Polymerase Chain Reaction (qRT-PCR) identified significant upregulation of AXL, a finding that was also confirmed by immunohistochemistry (IHC) and Western blotting in the PC9-GR2 cell line (data not shown).

Afatinib retained some inhibitory activity in the two PC9 gefitinib-resistant cells (PC9-GR1 and PC9-GR4) that have developed the T790M resistant mutation (Figure 1 and Table 1). None of the EGFR TKIs (afatinib, gefitinib or erlotinib) was active in the rest of the PC9 gefitinib-resistant clones, or in the PC9-ER cell line. Similarly, neither afatinib nor gefitinib were active in the H1650 cell line, which harbors the EGFR exon 19 deletion but has also a phosphatase and tensin homologue (PTEN) deletion [26] and particularly displays low expression of the BH3-only protein, Bcl-2 interacting mediator of cell death (BIM; also known as BCL2-like 11) [27, 28] (Figure 1 and Table 1).

### *In vitro* growth inhibition of EGFR mutant NSCLC cells treated with afatinib in combination with TPCA-1

**Figure 2:**
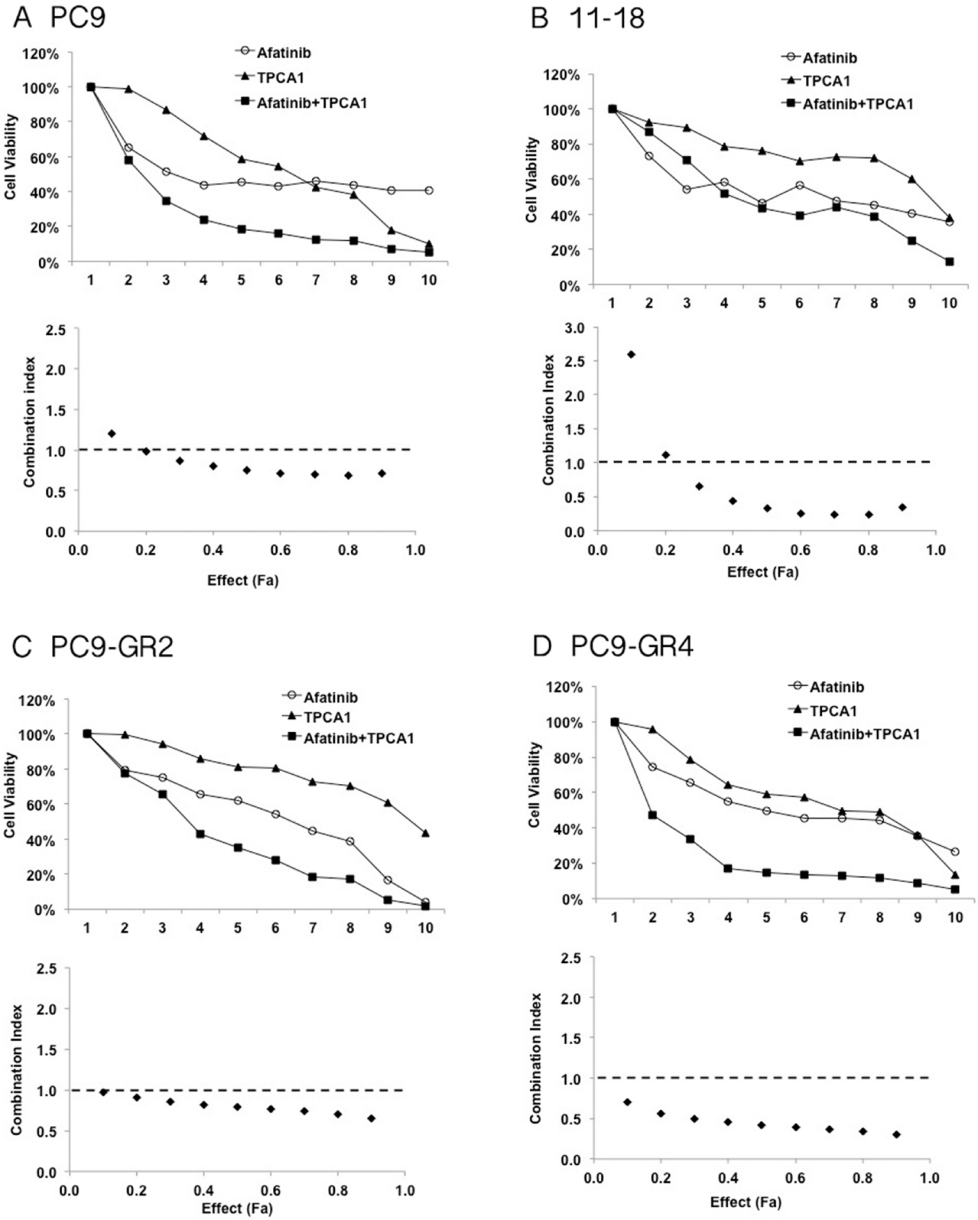
Effect of the double combination of afatinib and TPCA-1 in four EGFR mutant cell lines PC9 (**A**), 11–18 (**B**), PC9-GR2 (**C**) and PC9-GR4 (**D**) cells were treated with serial dilutions of afatinib and TPCA-1 alone and with their double combination for 72 h. The cell viability was measured by MTT and the synergy between the drugs was determined using the Chou and Talalay method (Chou and Talalay plot or Fa plot). The dotted horizontal line at 1 indicates the line of additive effect. Effect (Fa) indicates the fractional inhibition for each combinational index (CI). To calculate drug concentration for each Fa point the drugs were mixed using constant ratios corresponding to 1/8, 1/4, 1/2, 5/8, 3/4, 7/8, 1, 1.5 and 3 of the individual IC50 values for each drug in PC9, 11–18, PC9-GR2 and PC9-GR4 cells. The results represent the means of at least three independent experiments.

Based on previously reported knowledge that STAT3 activation can limit the cellular response to EGFR TKI treatment [13, 15, 18, 20], we assessed the growth inhibitory effects of the combination of afatinib plus TPCA-1 (STAT3 inhibitor) in EGFR mutant cell lines. We performed an MTT cell proliferation assay on EGFR TKI sensitive and resistant cells and we used the method of constant ratio drug combination proposed by Chou and Talalay [29] to determine synergy, additivity, or antagonism of afatinib plus TPCA-1. A 72-hour exposure to afatinib and TPCA-1 resulted in a clear synergism in PC9 cells as measured by the combination Index (CI) analysis, with a CI of 0.82 (Figure 2A). A clear synergism was also observed by adding TPCA-1 to afatinib in 11–18 cells with a CI of 0.69 (Figure 2B). Of interest the synergism was also evident in two PC9 gefitinib-resistant cells. Specifically, in PC9-GR2 cells, that do not harbor the T790M mutation, the combination of afatinib (in the IC50 dose of 4 μM) and TPCA-1 was synergistic with a CI of 0.80 (Figure 2C). In the PC9-GR4 cell line, that harbors the T790M mutation, the combination of afatinib and TPCA-1 was highly synergistic with a CI of 0.45 as shown by the isobologram analysis and the representative curves in Figure 2D. An additive effect was observed with the combination of afatinib and TPCA-1 in the H1975 cell line, with a CI close to one (CI = 0.92). These results indicate that combined treatment of EGFR mutant NSCLC cell lines with a STAT3 inhibitor and afatinib is associated with enhanced antitumor effect.

### STAT3 inhibition potentiates the effect of afatinib on downstream signaling pathways in EGFR mutant NSCLC cells

**Figure 3:**
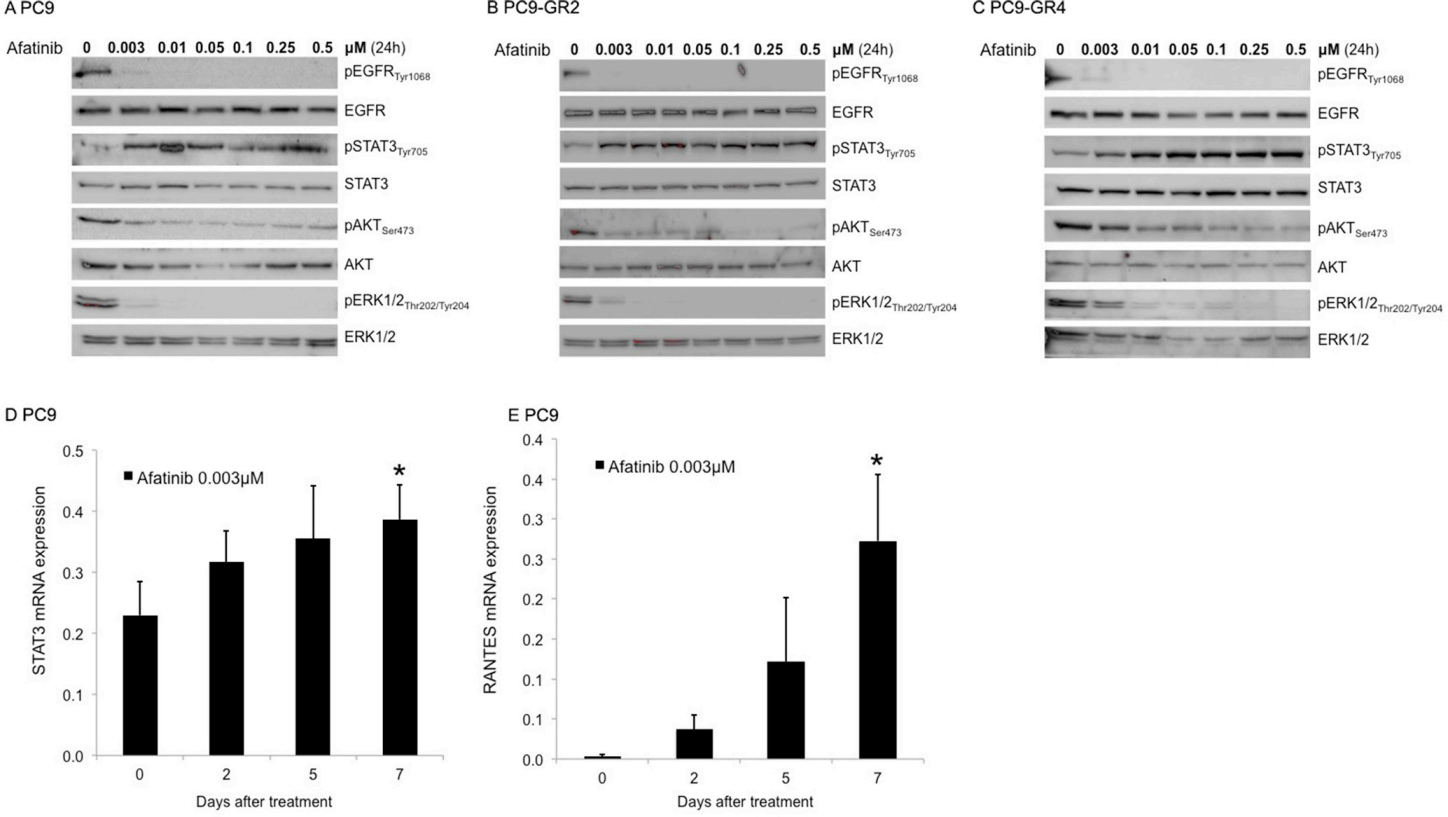
Effect of afatinib on signal transduction pathways Protein lysates from the PC9 (**A**) PC9-GR2 (**B**) and PC9-GR4 (**C**) cell lines treated with increasing doses of afatinib for 24 hours were collected and assessed by western blot analysis. STAT3 (**D**) and Rantes, (**E**) mRNA expression was measured using quantitative reverse transcription-PCR in PC9 cells that were treated with 0.003 μM of afatinib for seven days. Data were generated from a minimum of three replicates. β-actin was used to normalize gene expression. Data are presented as the means ± standard deviation; *P < 0.05.

**Figure 4:**
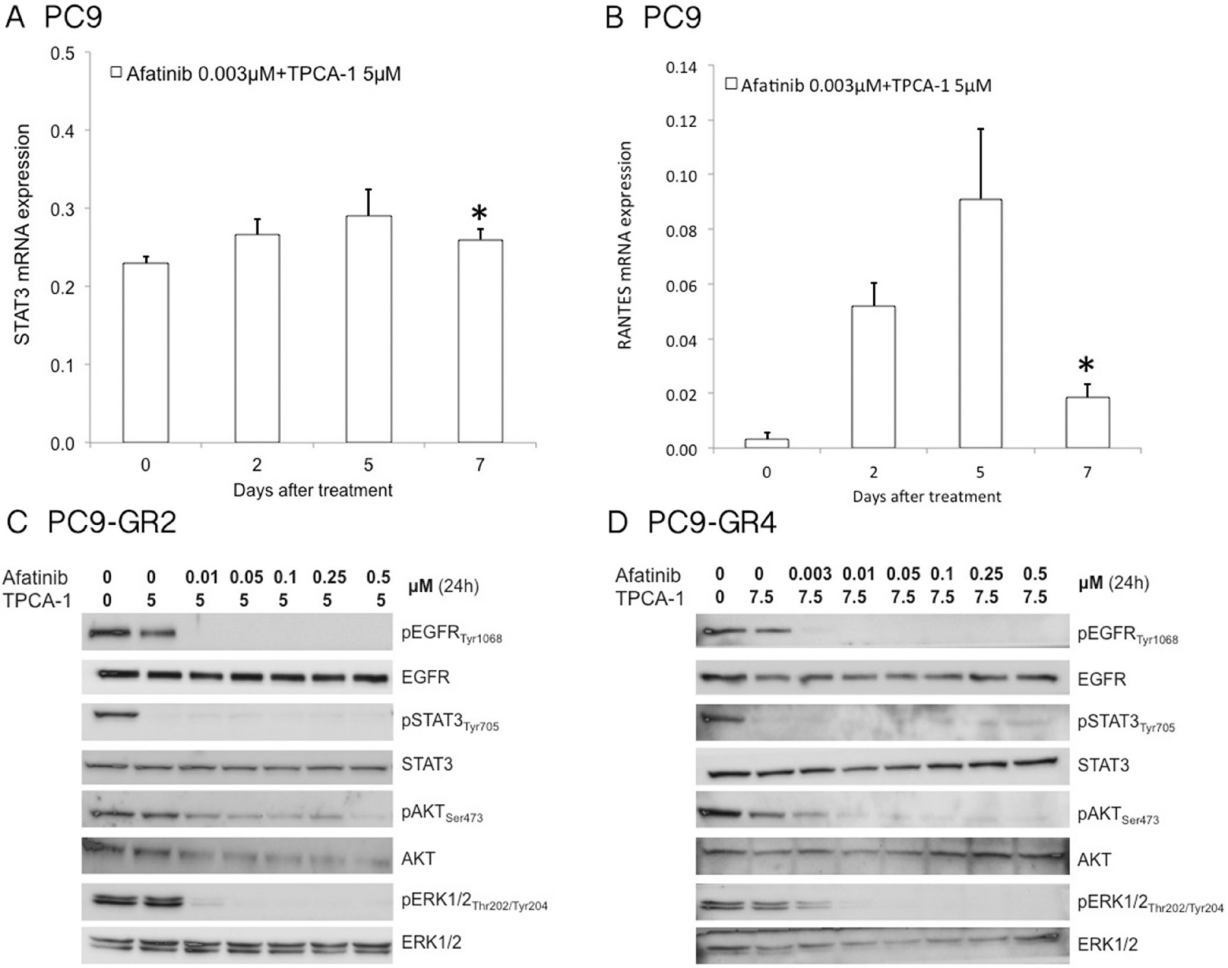
Effect of afatinib plus TPCA-1 on signal transduction pathways STAT3 (**A**) and Rantes, (**B**) mRNA expression were measured using quantitative reverse transcription-PCR in PC9 cells that were treated with 3.1M of afatinib and 2.5 μM of TPCA-1 for seven days. Data were generated from a minimum of three replicates. β-actin was used to normalize gene expression. Data are presented as the means± standard deviation; *P >0.05. Afatinib plus TPCA-1 inhibits phosphorylation of EGFR and STAT3 in a dose-dependent manner in PC9-GR2 (**C**) and PC9-GR4 (**D**) cells. Protein lysates from the PC9-GR2 and PC9-GR4 cell lines treated with increasing doses of afatinib and TPCA-1 for 24 hours were collected and assessed by western blot analysis.

We then tried to explore the molecular mechanism by which STAT3 inhibition with TPCA-1 enhances the efficacy of afatinib alone in EGFR mutant cell lines. To this scope, we first examined the phosphorylation status of EGFR, STAT3, AKT and ERK1/2 after afatinib treatment in the two afatinib-sensitive cell lines, PC9 and PC9-GR4 and the afatinib resistant cell line PC9-GR2. Afatinib suppressed EGFR, AKT and ERK1/2 phosphorylation but increased pSTAT3_Tyr705_ in a dose-dependent manner in all the cell lines (Figure 3A–3C). PC9 cells showed increased STAT3 and RANTES (regulated upon activation, normal T-cell expressed and presumably secreted) mRNA levels following seven days of afatinib treatment as examined by qRT-PCR analysis (Figure 3D–3E). RANTES expression is dependent on a transcription factor complex of STAT3 with NF-κB [30]. When afatinib was combined with TPCA-1 in the PC9 cell line, no increase in the mRNA expression of STAT3 or RANTES could be detected after seven days of treatment (Figure 4A–4B). Furthermore in the two PC9 gefitinib-resistant cells, PC9-GR2 and PC9-GR4, in which the double combination was highly synergistic, pSTAT3_Tyr705_ was clearly abrogated when TPCA-1 was added to afatinib (Figure 4C–4D).

### Afatinib treatment increases the fraction of ALDH^+^ cells

**Figure 5:**
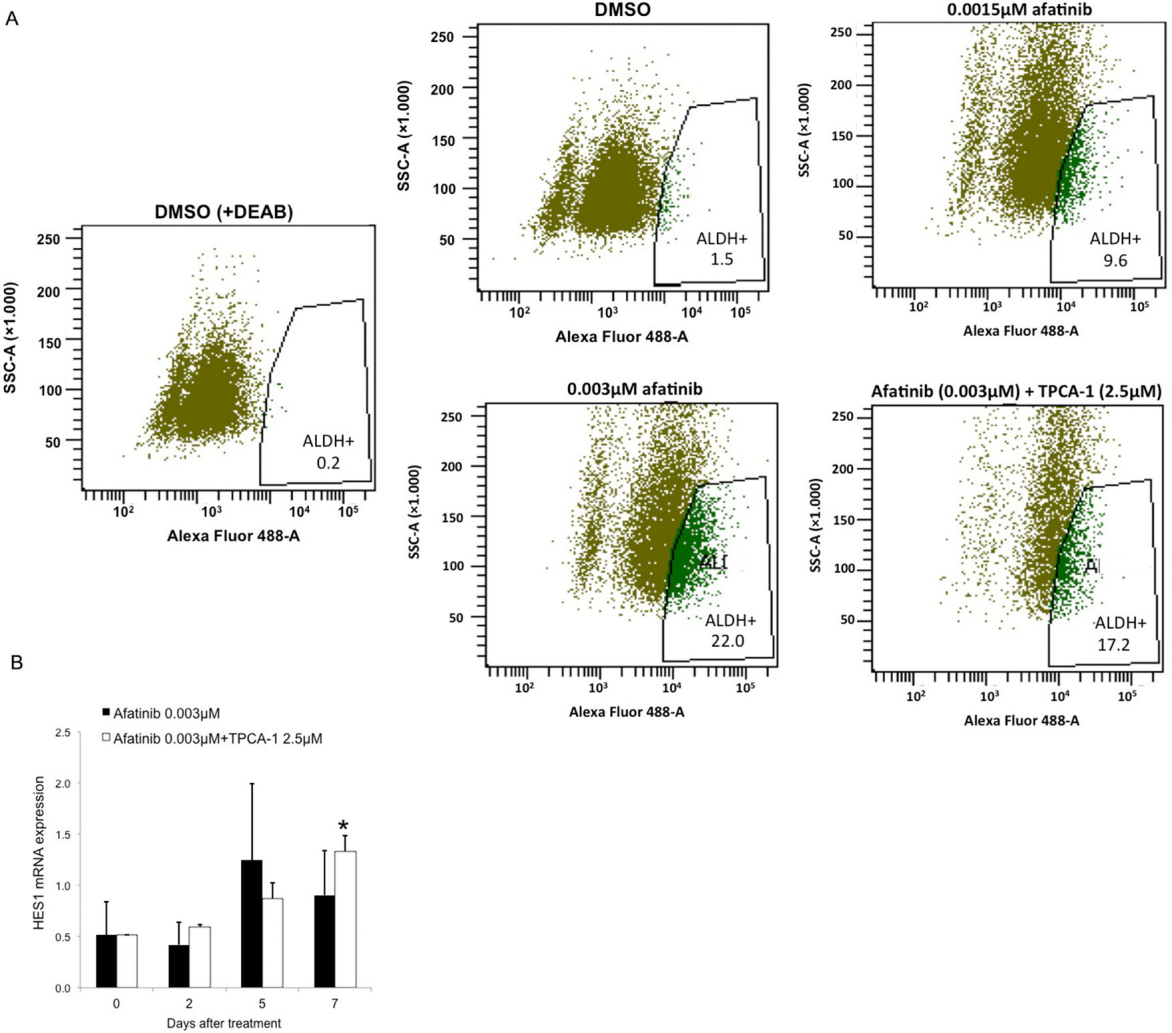
Afatinib treatment of EGFR-mutant lung cancer cells increases the fraction of ALDH+ cells in a dose-dependent manner (**A**) PC9 cells were treated with DMSO, and 0.0015 μM and 0.003 μM of afatinib or 0.003 μM of afatinib plus 2.5 μM of TPCA-1 for 7 days, and subjected to Aldefluor assay to detect the ALDH+ cells. A portion of the cells was preincubated with the ALDH inhibitor DEAB (+DEAB) to provide a gate (ALDH- cells) for flow cytometry. (**B**) HES1 mRNA expression were measured using quantitative reverse transcription-PCR in PC9 cells that were treated with 0.003 μM of afatinib or 0.003 μM of afatinib plus 2.5 μM of TPCA-1 for seven days. Data were generated from a minimum of three replicates. β-actin was used to normalize gene expression. Data are presented as the means ± standard deviation; *P < 0.05.

We then tested whether afatinib alone or in combination with TPCA-1 had an effect on the fraction and number of stem-like cells in the PC9 cell line. Cells were treated with DMSO or with 0.0015 μM and 0.003 μM of afatinib. Equal numbers of cells were subjected to ALDH enzymatic activity assays with the ALDH positive (ALDH^+^) and ALDH negative (ALDH^-^) cells quantified using flow cyrometry. There was a dose-dependent increase in the fraction of ALDH^+^ cells upon treatment with afatinib compared with DMSO treated cells (Figure 5A). Concomitant treatment with afatinib and TPCA-1 eliminated much of the ALDH^-^ population but the proportion of ALDH^+^ cells was not affected (Figure 5A). This observation indicates that the afatinib-induced increase of ALDH^+^ cells is independent of STAT3 modulation. The qRT-PCR analysis of Hairy and Enhancer of Split 1 (HES1), that functions in the maintenance of cancer stem cells, showed a significant increase after seven days of treatment with afatinib alone or in combination with TPCA-1 in PC9 cells (Figure 5B).

## DISCUSSION

Although EGFR TKIs induce a significant number of radiographic responses, almost no complete responses are obtained and the median PFS does not exceed more than one year. Still no combinatory therapies based on EGFR TKIs have been approved for the treatment of EGFR mutant NSCLC patients in first line. As soon as EGFR mutations were discovered in NSCLC, they were associated with dramatic response to gefitinib [31, 32]. It was also noted that EGFR mutant lung cancer cell lines selectively activate AKT and STAT3 signaling pathways, promoting cell survival. Importantly, NSCLC cells expressing mutant EGFR underwent extensive apoptosis after treatment with pharmacological inhibitors of AKT (Ly294002) and STAT (AG490) signaling [33]. The goal of the present study was to investigate whether STAT3 is activated almost immediately after afatinib treatment by tyrosine phosphorylation in response to epidermal growth factor (EGF) and interleukin-6 (IL-6) [34], similar to what has been reported for erlotinib or gefitinib therapy [13, 20]. EGFR mutations activate ERK, AKT and STAT3 directly or through IL-6-JAK2 [18, 33, 35]. pSTAT3_Tyr705_ is induced two hours after erlotinib or gefitinib treatment [13, 15, 20]. An IL-6 mediated crosstalk between NF-κB and STAT3 has been described in EGFR-mutant NSCLC cells [15]. We have recently shown that gefitinib or osimertinib treatment results in the activation of not only STAT3, but also Src-YAP1 signaling, potentially operating downstream of IL-6, to promote cell survival and limit the initial response to EGFR TKI treatment in EGFR mutant lung cancer [23].

Our findings indicate that abrogating STAT3 signaling is relevant in EGFR mutant cells, showing remarkable synergism when afatinib is combined with TPCA-1 (a STAT3 inhibitor) [15] in gefitinib-sensitive mutant EGFR cells (Figure 2A–2B), as well as in gefitinib-resistant PC9 cells, that are resistant to single afatinib treatment, particularly in PC9-GR2 (harboring AXL overexpression) (Figure 1 and Figure 2C). This observation is intriguing since we previously reported that increased AXL expression was a relevant mechanism of acquired resistance to EGFR TKIs [36]. The fact that the IC50 for afatinib in PC9-GR4 (38% allelic fraction of T790M) was in sub-nanomolar range with significant synergism in combination with TPCA-1 was quite noteworthy (Figure 2D). Although afatinib inhibits ERK and AKT signaling in PC9, PC9-GR2 and PC9-GR4, it does not abrogate pSTAT3_Tyr705_ (Figure 3A–3C). A gradual increase in STAT3 and RANTES mRNA levels was observed after seven days of treatment (Figure 3D–3E). This finding is of utmost importance since STAT3 activation can result in increased concentration of un-phosphorylated STAT3, driving a second wave of gene expression, such as RANTES [37]. Other studies have shown that inhibition of STAT3 phosphorylation leads to down-regulation of RANTES mRNA levels. However, the combination of afatinib with TPCA-1 was highly synergistic in PC9-GR4 cells, inhibiting pSTAT3_Tyr705_ and preventing up-regulation of STAT3 and RANTES mRNA levels (Figure 4). Finally we observed that afatinib increased the ALDH^+^ cell sub-population (Figure 5A). Although in other studies ALDH^+^ are reduced with IL-6/JAK1/STAT3 signaling blockade [38], we did not observe any decrease of ALDH^+^ cells with the combination of afatinib and TPCA-1 (Figure 5B). ALDH^+^ cells are considered representative of cancer stem-like cells [21] and are reported to be a consequence of crosstalk between EGFR and NOTCH signaling pathways in EGFR mutant tumors [22]. HES1 mRNA expression was elevated following therapy of PC9 cells with either afatinib or the combination of afatinib plus TPCA-1 (Figure 5C).

**Figure 6:**
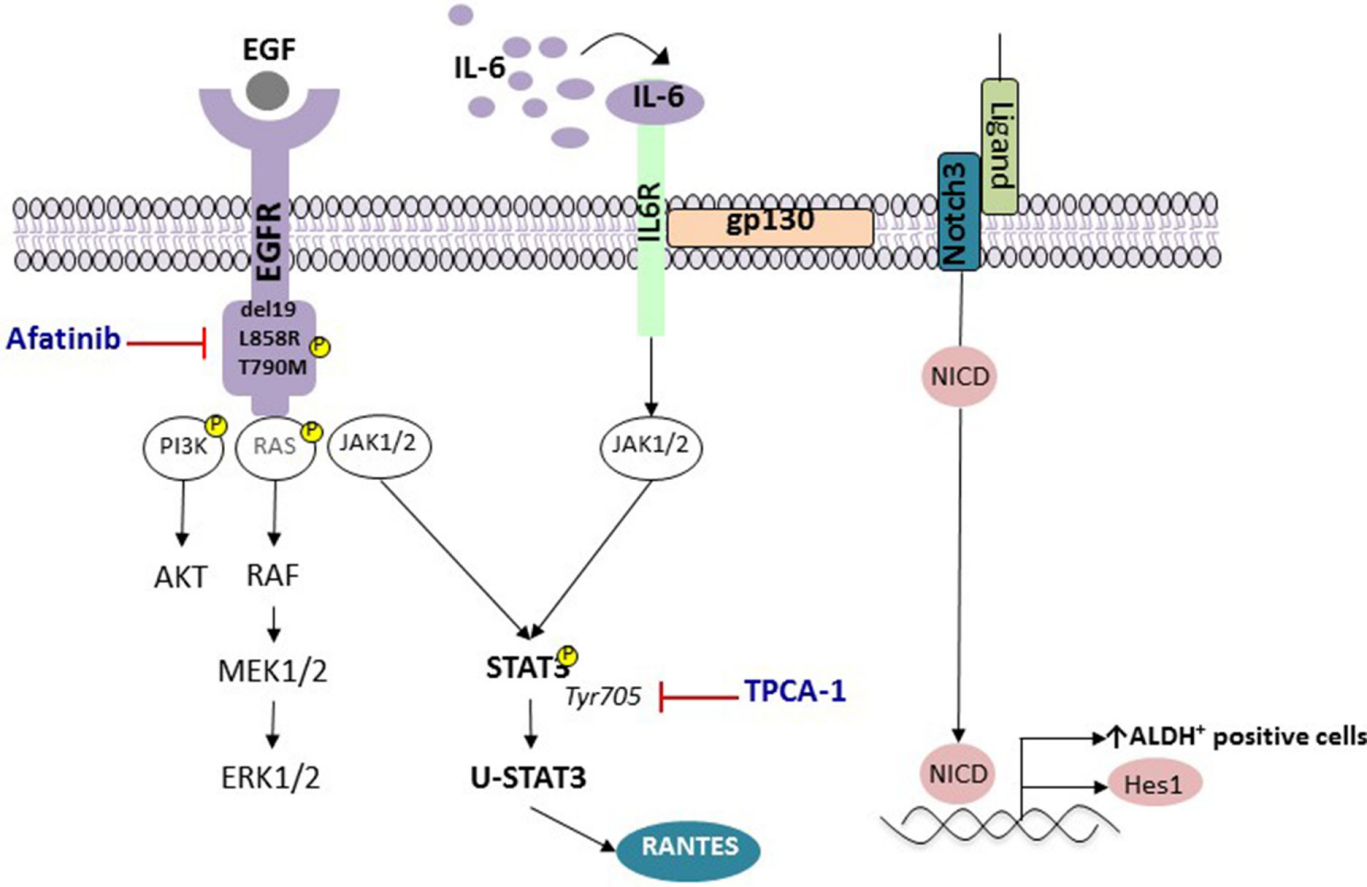
STAT3 activation and enrichment in ALDH+ cells in EGFR-mutant NSCLC Activation of EGFR has been linked to the pro-survival signaling pathways, including AKT, STAT3 and RAF/MEK/ERK mitogen-activated protein kinase (MAPK). EGFR mutant lung cancer cells produce high IL6 levels, which subsequently activates the gp130/JAK/STAT3 pathway. Unphosphorylated-STAT3 (U-STAT3), induced due to activation of the STAT3 in response to EGFR mutations or ligands such as IL-6, activates genes such as RANTES.

Accumulated evidence indicates that the combination of EGFR TKI with STAT3 inhibitors could be of benefit and increase the anti-tumor activity of these agents. In addition to TPCA-1, several repurposed and clinically approved drugs are considered active STAT3 inhibitors [39]. Clinical trials are warranted for the combination of afatinib plus STAT3 inhibitors. Figure 6 shows our working model. Although combinatory therapy with STAT3 inhibitors represents only an initial step towards progress in the management of EGFR mutant lung cancer, compelling evidence indicates the need for a switch from monotherapy to combination therapy and illustrates how pre-clinical research could pave the way to implement such clinical trials.

The incidence of the acquired EGFR T790M mutation is as prevalent in patients treated with afatinib as in patients treated with erlotinib or gefitinib [40]. Even though afatinib cannot clinically reverse T790M-mediated resistance, the evidence that afatinib inhibits cell proliferation in PC9-GR4 with T790M, as well as in PC9-GR2 with AXL overexpression is in line with its ability to delay emergence of resistance through this mechanism. Indeed, gene dosage of the T790M EGFR mutation seems to be important [41]. Evaluation of this combination is thus of interest and merits further investigation, especially now that we are on the verge of monitoring EGFR mutations in circulating DNA [17, 42]. The early detection of T790M in plasma could be of further assistance in EGFR mutant patients treated with afatinib in combination with STAT3 inhibitors.

## MATERIALS AND METHODS

### Chemicals and reagents

EGFR inhibitors (erlotinib, gefitinib and afatinib) and TPCA-1 were purchased from Selleck Chemicals (Houston, TX, USA). Drugs were prepared in dimethyl sulfoxide (DMSO) at a concentration of 10–100mmol/L stock solutions and stored at –20 °C. Further dilutions were made in culture medium to final concentration before use. Phospho-EGFR (Tyr1068), phospho-STAT3 (Tyr705), phospho-ERK1/2 (Thr202/Tyr204) and β-actin antibodies were purchased from Cell Signaling Technology (Beverly, MA). The secondary antibodies Amersham ECL-anti-rabbit IgG horseradish peroxidase-linked species-specific whole antibody and Amersham ECL-anti-mouse IgG peroxidase-linked whole antibody were purchased from GE Healthcare UK limited (Buckinghamshire, UK).

### Cell lines

Human lung adenocarcinoma PC9 cells, harboring EGFR exon 19 deletion (E746-A750) were provided by F. Hoffmann-La Roche Ltd. with the authorization of Dr. Mayumi Ono (Kyushu University, Fukuoka, Japan). Human lung adenocarcinoma 11–18 and H3255 cells, harboring EGFR exon 21 L858R mutation, were provided by Dr. Mayumi Ono and Dr. Daniel Costa (Department of Medicine, Harvard Medical School, Boston, MA) respectively. Human lung adenocarcinoma H1975 cells, harboring both sensitizing L858R and resistant T790M mutation, were purchased from the American Type Culture Collection (ATCC). Human lung adenocarcinoma H1650 cells, harboring EGFR exon 19 deletion (E746-A750) and PTEN loss, were purchased from the American Type Culture Collection (ATCC). We have generated six EGFR TKI-resistant cell lines by treating EGFR TKI-sensitive PC9 cells with increasing concentrations of gefitinib (GR1-5) or erlotinib (ER). Sequencing analyses revealed that all six cell lines retained the EGFR exon 19 deletion (Table 1), while the T790M mutation emerged in two (PC9-GR1 and PC9-GR4 at allelic fractions of 25 and 38% respectively). All cell lines were maintained in RPMI (Roswell Park Memorial Institute medium) 1640 supplemented with 1x penicillin/ streptomycin/ glutamine (Gibco) and 10% fetal bovine serum (FBS) (Gibco) in 5% CO2, 37 °C cell culture incubator and were routinely evaluated for mycoplasma contamination.

### Cell viability assay

Cells were seeded on 96-well plates at the following densities: PC9 at 2 × 10^3^, 11–18 at 3 × 10^3^, H3255, PC9-GR2, PC9-GR4 at 4 × 10^3^ and H1975 at 6 × 10^3^ and incubated for 24 hours. Next, the cells were treated with serial dilutions of the drugs administrated at doses typically corresponding to 1/8, 1/4, 1/2, 5/8, 3/4, 7/8, 1, 1.5 and 3 of the individual IC50 values. After 72 hours of incubation, 0.275 mg/ml of MTT reagent (Sigma Aldrich) was added to the medium in the wells for 2 hours at 37°C, formazan crystals in viable cells were solubilized with 100 ml DMSO and spectrophotometrically quantified using a microplate reader (Varioskan Flash Thermo Electron) at 550 nm of absorbance. Fractional survival was then calculated by dividing the number of cells in drug-treated wells by the number of cells in control wells. Data of combined drug effects were subsequently analyzed by the Chou and Talalay method [29]. CI values < 1, =1 and > 1 indicated synergism, additive effect and antagonism, respectively.

### Gene expression analyses

RNA was isolated from the cell lines in accordance with a proprietary procedure (European patent number EP1945764-B1) as previously described [43]. The primer and probe sets were designed using Primer Express 3.0 Software (Applied Biosystems) according to their Ref Seq (http://www.ncbi.nlm.nih.gov/LocusLink). Specifically, sequences of the primer sets and the probes for amplification of the genes examined in the study were as follows: STAT3, forward primer, 5′-CACCTTCAGGATGT CCGGAA-3′, reverse primer, 5′-ATCCTGGAGATTCTCT ACCACTTTCA-3′, probe 5´-FAM AGAGTGCAGGATC TAGA-MGB 3′; RANTES, forward primer, 5′-CATC TGCCTCCCCATATTCCT-3′, reverse primer, 5′-AGTGGG CGGGCAATGTAG 3′, probe 5´-FAM ACACCACACCC TGCTG-MGB 3′; HES1, forward primer, 5′-GGACATT CTGGAAATGACAGTGAA-3′, reverse primer, 5′-CAG CACACTTGGGTCTGTGC-3′, probe 5´-FAM ATGAC GGCTGCGCTGA-MGB 3′; β-actin, forward primer, 5′- TG AGCGCGGCTACAGCTT-3′, reverse primer, 5′-TCCTT AATGTCACGCACGATTT-3′, probe 5´-FAM ACCACCA CGGCCGAGCGG TAMRA 3′. Quantification of gene expression was performed using the ABI Prism 7900HT Sequence Detection System (Applied Biosystems). Expression levels were calculated according to the comparative ΔΔCt method. Commercial RNA controls were used as calibrators (Liver and Lung; Stratagene, La Jolla, CA, USA). For each cell line, three independent experiments were performed.

### Western blotting

Cells were washed with cold phosphate-buffered saline (PBS) and re-suspended in ice-cold radio-immunoprecipitation assay (RIPA) buffer (20 mM Tris- hydrochloric acid in pH 7.5, 1% Nonidet P-40, 0.5% sodium deoxycholate, 150 mM sodium chloride, 1 mM ethylenediaminetetraacetic acid, 1 mM EGTA, 2.5 mM sodium pyrophosphate, 1 mM beta-glycerophosphate and 1 mM sodium vanadate) containing protease inhibitor mixture. Following cell lysis and centrifugation at 14000 × rpm for 10 min at 4°C, the resulting supernatant was collected as the total cell lysate. Briefly, the lysates containing 30 μg proteins were electrophoresed on 10% SDS-polyacrylamide gel electrophoresis (Life Technologies) and transferred to polyvinylidene difluoride membranes (BIO-RAD laboratories). Membranes were blocked in Odyssey blocking buffer (LI-COR Biosciences). All target proteins were immunoblotted with appropriate primary and peroxidase conjugated secondary antibodies. Quimioluminiscence bands were detected with Bio-Rad ChemiDoc MP Imaging System.

### Aldefluor assay and flow cytometry

The Aldefluor assay kit (Stem Cell Technologies, Vancouver, BC, Canada) was used to determine the profile of cells with high and low ALDH activity. The assay was performed according to manufacturer's instructions, with certain modifications. Briefly, 2 × 10^6^ cells were suspended in Aldefluor assay buffer and divided into two groups. One group was pretreated for 10 min with the ALDH-specific inhibitor diethylaminobenzaldehyde (DEAB) and then both groups were incubated with ALDH enzyme substrate BODIPY-aminoacetaldehyde for 30 min at 37°C. Cells were then centrifuged and re-suspended in a fresh Aldefluor assay buffer to remove the unutilized substrate. The fluorescence intensity of stained cells was analyzed using a FACS canto II (BD Biosciences) flow cytometer. For the analysis, DEAB-treated sample was used as a negative control. The ALDH activity of a sample was determined to be ‘high’ or ‘low’ based on fluorescence intensity above or below the threshold defined by the reaction with DEAB.
